# Network Adaptation Improves Temporal Representation of Naturalistic Stimuli in *Drosophila* Eye: I Dynamics

**DOI:** 10.1371/journal.pone.0004307

**Published:** 2009-01-30

**Authors:** Lei Zheng, Anton Nikolaev, Trevor J. Wardill, Cahir J. O'Kane, Gonzalo G. de Polavieja, Mikko Juusola

**Affiliations:** 1 Department of Biomedical Science, University of Sheffield, Sheffield, United Kingdom; 2 Department of Genetics, University of Cambridge, Cambridge, United Kingdom; 3 Department of Theoretical Physics, Universidad Autónoma de Madrid, Madrid, Spain; 4 Instituto ‘Nicolás Cabrera’ de Física de Materiales, Universidad Autónoma de Madrid, Madrid, Spain; 5 State Key Laboratory of Cognitive Neuroscience, Beijing Normal University, Beijing, China; University of Southern California, United States of America

## Abstract

Because of the limited processing capacity of eyes, retinal networks must adapt constantly to best present the ever changing visual world to the brain. However, we still know little about how adaptation in retinal networks shapes neural encoding of changing information. To study this question, we recorded voltage responses from photoreceptors (R1–R6) and their output neurons (LMCs) in the *Drosophila* eye to repeated patterns of contrast values, collected from natural scenes. By analyzing the continuous photoreceptor-to-LMC transformations of these graded-potential neurons, we show that the efficiency of coding is dynamically improved by adaptation. In particular, adaptation enhances both the frequency and amplitude distribution of LMC output by improving sensitivity to under-represented signals within seconds. Moreover, the signal-to-noise ratio of LMC output increases in the same time scale. We suggest that these coding properties can be used to study network adaptation using the genetic tools in *Drosophila*, as shown in a companion paper (Part II).

## Introduction

How do retinal neurons adapt to best encode environmental light changes to neural responses? Because the capacity of eyes to capture, process and transmit information is limited, there is an expectation of efficiency for retinal coding [1], [2]. Here, the use of information theory [3] has helped to formalize such ideas by predicting efficient models for two important coding problems: how to shape (i) the *static* nonlinear input/output relations, *i.e.* contrast or characteristic gain, of visual neurons [4] and (ii) their *dynamic* filtering properties [5]–[11] in order to maximize the information flow of naturalistic light stimuli.

The efficient representation of visual information [1], [2] requires matching of the coding strategy of neurons to the statistical structure of their stimuli so that the information carried by neural responses is maximized [2], [4], [11], [12]. It implies that neurons should strive to utilize their output range equally in different situations, since a message, in which every symbol is transmitted equally often, has the highest information content [3], [13]. Accordingly, retinal adaptation should improve coding efficiency by using the regularity and scale invariance of contrasts and other visual features in the natural scenes [4], [8], [10], [14]. After learning the probabilities of encountering such features, optimal adaptation would then remove any redundancy in the neural output, whilst allocating increased representation to frequently encountered features [15]. Experimental tests of this equalization have been performed, for instance, in the LMCs of the blowfly eye [4], and in visual relay neurons of thalamus [16]. These studies concentrated on stationary statistics at bright stimulus conditions (high signal-to-noise ratio). However, the statistics of natural stimuli are nonstationary and retinal neurons therefore need to adapt continuously to the current statistics [8], [17], [18].

When animals or their eyes move, images of natural scenes projected on the retinas can change greatly [11]. Changing solar elevation and weather conditions generate logarithmic luminance changes; even the reflectance differences within sunlit scenes can vary over 10^4^-fold [17]. While bright scenes can overwhelm photoreceptors with redundant information [1], [2], in a dim environment (low signal-to-noise ratio) there is little light information to gather within behaviorally relevant integration times, and vision becomes unreliable [19]. The problem of a vast dynamic intensity range is partially solved by retinal neurons encoding contrast that is independent of the level of illumination, but the problem of noise still requires adaptation to changing statistics [20]. What are the general coding strategies for this dynamic optimization?

To characterize the general features how retinal neurons encode naturalistic stimuli at different luminance levels, van Hateren deduced spatiotemporal filters that maximized transmission of information through a noisy channel of limited dynamic range [11]. The filters mimic active gain control of retinal neurons; integrating at dim illumination (increasing output redundancies), and differentiating at bright illumination (reducing output redundancies). He compared them against the responses of Large Monopolar Cells (LMCs) of the blowfly eye to flashed stimuli in various light backgrounds (*mean* adaptation), and found that the filters approximated the neural outputs. Since then different statistical and information theoretical methods have been used to quantify coding in insect photoreceptors for various dynamic stimuli, including *variance* and *speed* adaptation [6], [17], [21]–[29].

However, retinal adaptation can be difficult to quantify in behaviorally relevant time scales. For animals to gather enough information about the stimulus to execute behavior or make a successful choice, integration times can span from ∼200 ms to seconds [30], [31]. Thus, we need to complement statistical and dynamical studies [6], [20], [24]. To partially overcome this problem, we devised a strategy of repeating a brief naturalistic stimulus pattern (from 100 ms to 1 s) to retinal neurons while collecting their voltage responses. We then analyzed neural adaptation to these stimuli at time scales relevant for behavioral decisions.

**Figure 1:**
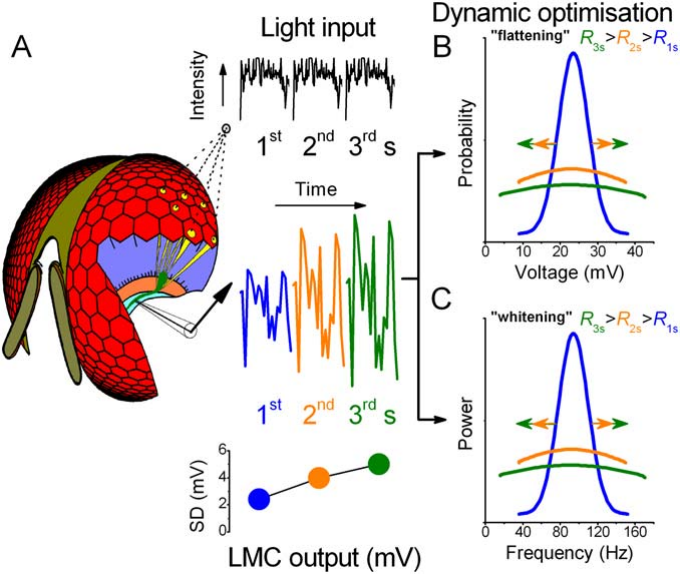
Theories of dynamic optimization of early neural responses by adaptation. A. Neurons in lamina (orange section in the opened eye) generate responses to a naturalistic light pattern, which is repeated at the centre of mutual receptive field (gray circle) of six photoreceptors (R1–R6, yellow) and visual interneurons, Large Monopolar Cells (LMCs, one shown in green). These cells sample light information from the same small area in space (dotted lines). By recording intracellularly from LMCs, the quality of synaptic output can be assessed *in vivo*. When the light input pattern (local statistics) is reencountered (repeated), the prior experience of this R-LMC-R system [25], [32], [34] (named such because of its synaptic feedforward and feedback connections) should improve its voltage responses (blue, orange and green traces) over time. Note how the size of the responses, and thus, SD increases, as wider distributions equal greater sensitivity. This could happen during adaptation in two ways, as shown in panels B and C. B. the responses increase, “flatten” their probability distribution (PDF; blue = 1^st^, orange = 2^nd^, green = 3^rd^ s). C. In the frequency domain, changes in the speed of the responses “whiten” their power. Such redistributions of synaptic output improve the neural information transfer rate, *R*, over time (*R*_3 s_>*R*_2 s_>*R*_1 s_).

An efficient coding system would use its full amplitude and frequency ranges to code for incoming signals. We therefore predict that adaptation would serve to adjust these ranges to the changing statistics of light input. After a transition between two stimuli with different statistics (such as dark-light transition), adaptation would adjust the system's output continuously to best represent the temporal structure (or local statistics) of the new stimulus. Here we investigate how *Drosophila* photoreceptors (R1–R6) and LMCs, whose graded voltage responses are shaped by a web of feedforward and feedback synapses [25], [32]–[35], adapt together in order to best encode information about temporal patterns in naturalistic light stimuli. Functionally, this system thus can be viewed as R-LMC-R [25], [36] (the cells form a processing unit in which information travels both forward and backward), and much of its adaptation considered as network adaptation [36]. By using a protocol of repeated stimulus patterns and recording intracellularly from R1–R6s and LMCs (Fig. 1A), we observe that the amplitude distribution of voltages “flattens” and that frequency spectra “whiten” over time (Fig. 1B, C). Thus, one would predict that as the system's sensitivity to local statistics increases, the distribution of its output, and accordingly its SD (Fig. 1A), should widen. Experimentally, we find this to be the case for the LMC output.

We show that adaptation makes coding of visual information in LMC output more efficient within seconds. It improves sensitivity to signals, which were initially under-represented in the first response to a novel stimulus pattern, when encountering this pattern again. While this improvement follows different time constants for different luminance levels, it increases the signal-to-noise ratio in the LMC output about the naturalistic stimulus over repeated presentations. We further show that this encoding is insensitive to pattern speed, and needs little re-sensitization.

In this paper, we quantify how adaptation shapes neural encoding of local stimulus statistics in the *Drosophila* R-LMC-R system, the consequences of which were not known before in this system. In a companion paper [36], we have used the genetic tools of *Drosophila* to show how synaptic feedforward and feedback mechanisms in the R-LMC-R system result in this form of efficient coding of naturalistic light stimulation.

## Results

Adaptation occurs continuously in the R-LMC-R system as the world projects onto the eyes of a behaving fly, but it is not clear how to best quantify the neural responses so that the underlying encoding strategies become clear. Our approach here was to repeatedly present a rich naturalistic contrast pattern with one second duration to the centre of the receptive field of a single photoreceptor or LMC. We then analyzed the evolution of their responses with millisecond time-resolution for each consecutive one second observation window. The stimulus contained 10,000 intensity values with approximate 1/*f* statistics [8], [17], *i.e.* it dominantly represented low stimulus frequencies. While such stimulation from a fixed point in space ignores spatial processing normally performed by LMCs [11], it benefits the following analysis by simplifying the R-LMC-R system into a single processing unit that lacks lateral communication from the neighboring systems. As the statistics and signal-to-noise ratio of the input are the same for each window, we obtain a continuous account of how these neurons use prior experience to readjust their output.

### Adaptation changes neural encoding of repetitive stimulus with luminance

**Figure 2:**
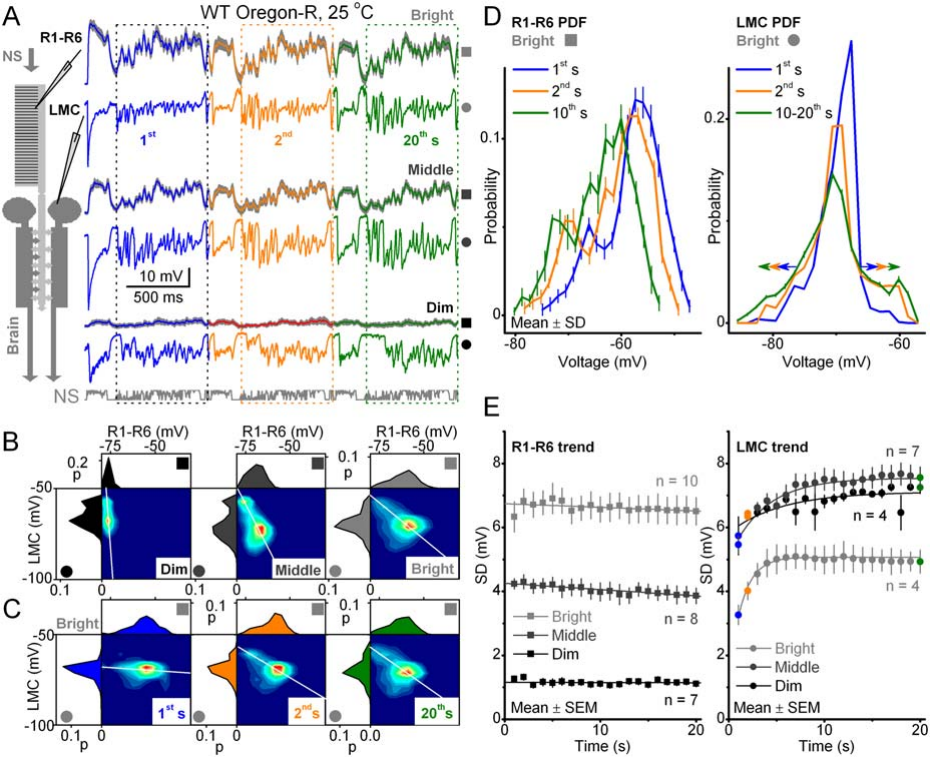
Adaptation changes neural encoding of repetitive naturalistic stimulus with luminance and time. Voltage responses of R1–R6 photoreceptors and LMCs to a repeated naturalistic stimulus pattern, NS, adapt with light intensity and over time. A. Responses of photoreceptors (mean±SD, n = 7) and a representative LMC to a 1-s stimulus, during the first stimulus repetitions at different luminance levels. Note that both the photoreceptors and LMC change their output to the same stimulus, including their maxima (peak responses), over luminance and time. Note also the contrast patterns that evoke the peak responses are different for the 1^st^, 2^nd^ and 20^th^ s of stimulation. B. The corresponding probability density functions (PDFs) for R1–R6s (top) and LMCs (left) and the joint probability density functions, calculated from the first 20 responses. jPDFs are shown as contour plots, in which hot colors denote high probability. The jPDFs quantify the input-output transformations, characterizing the synaptic throughput for the given luminance of stimulation. The white lines approximate most probable synaptic gains. C. PDFs and jPDFs are shown for the 1^st^, 2^nd^ and 20^th^ s of the bright stimulation. Note that the synaptic gain changes over time, highlighted by the inclination of the white lines. Although the synaptic gain changes over time, the photoreceptor signal changes very little, indicating that most adaptation in the phototransduction occurred within the first second. D. High resolution PDFs at different times during the bright stimulation show how adaptation changes photoreceptor and LMC outputs dynamically. PDFs of photoreceptors (left) remain rather intact, while PDFs of LMCs (right) flatten and widen over time (arrows) (*cf.* Fig. 1C). E. The time-dependent trends of adaptation in the PDFs are also seen in the SDs of the responses for each experiment (SDs are from the boxed data, 201–1000 ms in A). Desensitization of photoreceptors output (SDs, left) and sensitization of LMCs output (SDs, right) are fitted by lines or exponentials, respectively (*cf.* Fig. S1). LMCs: dim, τ_1_ = 5.42 s; middle, τ_1_ = 3.74 s; bright, τ_1_ = 1.38 s.

We recorded intracellular voltage responses of *Drosophila* photoreceptors and LMCs to the repeated naturalistic stimulus [24], [25] at different luminance levels (Fig. 2A), *in vivo*. To keep the state of adaptation comparable, the cells were dark-adapted for ∼30 s before first presenting the stimulus at each luminance level. In this paper, we present adaptation dynamics of photoreceptor and LMC output of WT flies at three luminance levels: dim (1,850), middle (60,000) and bright (1.85×10^6^ photon/s), as calibrated from voltage responses of photoreceptors to single photons [25]; see also (Fig. S1).

Photoreceptors produced faster and larger depolarizations in response to brighter stimuli. In LMCs, histamine-gated chloride channels [37] translated these responses to graded and phasic hyperpolarizations. At brighter luminance levels the output of LMCs became increasingly transient, peaking before the photoreceptor voltages peaked. For the bright stimulus at 25°C, the time-to-peak of voltage responses in photoreceptors was 47.2±16.7 ms (n = 10 cells) and 14.6±3.3 ms in LMCs (n = 12 cells, mean±SD, p<10^−5^ in Bonferroni-test for means).

From a theoretical point of view [11], the more transient LMC output is somewhat expected; when the signal-to-noise ratio of stimulus increases, the R-LMC-R system should adapt from a slow integrator to a fast differentiator to maximally package light information into voltage responses through a bandwidth-limited membrane [11]. However, we did not want to commit to any coding variables when analyzing the transformations from photoreceptors to LMCs. For example, because of the noncausality in the peak times (above), synaptic gain between the photoreceptor input and LMC output cannot be estimated simply by comparing their maximum amplitudes, although this simplified analysis has been used before [38], [39]. To provide the least biased analysis of the communication between these neurons, we chose to pair the photoreceptor and LMC voltages for each time-bin (or sampling point), and compare their transformations continuously. For any given window of time, we can quantify “input-output” transformations by counting the occurrences of voltage pairs across the whole “input-output” range of the system. We display the probabilities as a map by using relatively coarse binning (3.3 mV) as this increases pooled samples and reduces timing jitter, thereby giving a smooth representation of the recorded “input-output” dynamics (Figs. 2B–C).

Analysis of joint photoreceptor and LMC statistics during the first 20 s of dim-, middle- and bright-intensity stimuli showed that the strategy of photoreceptors and LMCs was to increase their amplitude ranges with brightening stimulation (Fig. 2B). The most probable photoreceptor-LMC voltage pairs provided an estimate of the synaptic gain during the given stimulation. The gain varied dynamically with the stimulus at each luminance; rather than being a simple static nonlinearity, *i.e.* a curve, it covered large pear-shaped areas in these “input-output” maps. A given photoreceptor input led to a range of different LMC output values, and a given LMC output could result from different photoreceptor values. Nonetheless, in agreement with blowfly synapse data [27], the changes in the joint probabilities demonstrated that synaptic gain adapted with light intensity, being highest (steepest slope of LMC/R activity) in dim and lowest in bright stimulation, presumably to encode LMC's representation of the given stimulus over a relatively regular voltage range. Thus, as the environmental light intensity scales logarithmically, the gain of R-LMC-R system changes accordingly to prevent saturation, and possibly also to work toward contrast constancy; see also [17], [20], [25].

### Adaptation changes neural coding of repetitive stimulus with time

On close inspection, the statistics of the joint photoreceptor and LMC responses are *non-stationary*. For example, compare the 1^st^, 2^nd^ and 20^th^ s of repeated stimulation at bright luminance level (Fig. 2C). Adaptation, acting within the first second, reduced the voltage output of photoreceptors, redistributing their responses over a narrower voltage range. This trend continued, although less prominently, with further stimulus repetitions. Thus, adaptation dynamics in the *Drosophila* photoreceptors mirrored those seen in blowfly photoreceptors under similar naturalistic stimulation [24]. On the other hand, in LMCs, adaptation caused a significant increase in the amplitude range of their voltage responses, spreading their probability distributions. Together these intensity- and time-dependent adaptation components changed the synaptic gain continuously.

### Adaptation shapes amplitude distributions dynamically

The ideal representation of light contrasts uses the widest available range of signal amplitude (“flattening”), as this provides the richest combination of patterns for the transmission [4], [40]. Figure 1B shows how a system could approximate such coding scheme. The optimal case for a channel constrained by fixed limits is to use every amplitude equally often. On the other hand, a Gaussian distribution is optimal for output from a channel constrained by a fixed response variance [3], [17]. LMC output probably faces both constraints, set by the reversal potentials of ions and by power dissipation when the membrane voltage is driven up and down to encode light stimuli [17]. For naturalistic light intensity series lasting minutes, the output of blowfly LMCs approximates a Gaussian distribution [17]. Here, for the much briefer stimuli at different luminance levels, the LMC output could not achieve a Gaussian distribution (Fig. 2B–C). Perhaps encoding was suboptimal because the distribution was not flat either [3], [13], which can happen when there are metabolic, time or processing constraints or noise exists [41], [42].

More important, however, was the observed increase in the amplitude distribution of LMC output with time (Fig. 2D), which suggests that neural encoding of the temporal structure of the light pattern, *i.e.* local statistics, was improving with each stimulus presentation. Photoreceptor (left) and LMC (right) probability density functions (PDFs) for dim, middle and bright stimuli (shown for bright), and the standard deviations of these distributions (Fig. 2E), for the first 20 s of stimulation, illustrate how the LMC output range expanded while the photoreceptor output range was compressed slightly. These two processes, photoreceptor desensitization and LMC sensitization, occurred with different time-courses but always accelerated with brightening.

To recapitulate, adaptation to a repetitive naturalistic stimulus enabled LMCs to generate larger voltage responses from the diminishing voltage responses of photoreceptors. Therefore, the equalization of LMC output must have reflected events occurring in the R-LMC-R system after light-adaptation in photoreceptors, *i.e.* post-phototransduction dynamics. Although the equalization seemed sub-optimal, *i.e.* the distribution of voltage responses was neither Gaussian nor flat, it suggested that the neural encoding is improving over time. In the following sections, we present similar results in the frequency domain, and importantly, that the signal-to-noise ratio of LMC output increases in time. These findings imply that adaptation improves the efficiency to code naturalistic light changes within seconds.

### Adaptation shapes frequency spectra dynamically

To maximize information transmission to the brain, the early sensory signals should also be coded with minimal correlation between them, using the available frequency range of the neurons [10], [11], [13]. Figure 1C illustrates the concept of “whitening”. For the optimal case, *i.e.* for a message of the highest information content, every temporal frequency would be used equally often. How are the changes in the photoreceptor and LMC outputs, which are seen as dynamic desensitization and sensitization, respectively, distributed over their limited frequency ranges at different luminance levels?

**Figure 3 pone-0004307-g003:**
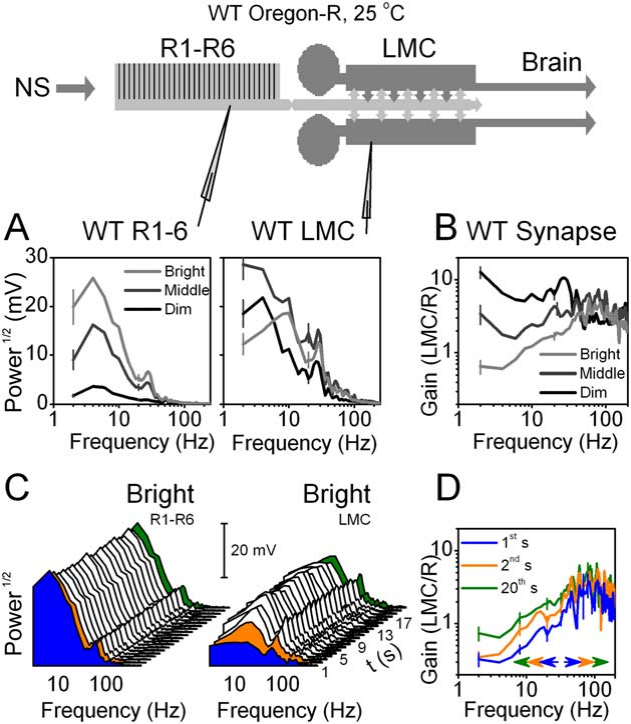
Adaptation shapes frequency spectra dynamically. Frequency spectra of photoreceptor and LMC voltage responses to repeated presentations of naturalistic stimulus, NS, vary with light intensity and over time. A. Mean frequency spectra of seven photoreceptors (left) and a characteristic LMC (right) for the first 20 s of dim, middle and bright stimulation. B. Corresponding synaptic gain changes with light intensity. Notice the progressive removal of low frequency signals with brightening luminance levels. C. Changes in photoreceptor (left) and LMC (right) frequency spectra to the repeated bright stimulus during the first 20 s (1st s = black; 2nd s = red; 20th s = green); adaptation affects mostly LMC frequency spectra in the five first seconds of repeated stimulation. D. Because of the increasing low frequency content (up arrows), synaptic gain spreads more evenly within the bandwidth over time (arrows). Error bars are SD.

The frequency spectrum of the voltage responses of photoreceptors and LMCs to dim-, middle- and bright-intensity stimulation (Fig. 3A) had characteristic 1/frequency behavior of the stimulus, *i.e.* low frequency components dominated the responses (see Materials and Methods) [8], [17]. The spectra had peaks at 10, 40 and 85 Hz, which can further serve as useful landmarks to facilitate their comparisons. Although the amplitude and bandwidth of both the photoreceptor and LMC outputs increased with light intensity, the LMC values were higher. Photoreceptors dedicated most of their voltage range to follow bright stimulation up to 40 Hz, while LMCs could still represent with reasonable voltage values the last landmark frequency at 85 Hz. The boosted frequency-range in the LMC output reflected adaptive filtering of the synaptic throughput. Figure 3B shows the frequencies, which were amplified by the synapse, as the ratio of the LMC and photoreceptor frequency spectra. For dim stimulation the synapse showed low-pass behavior, but with brighter stimulation, this shifted toward band-pass by transmitting higher frequencies [11], [27]. These changes were dynamic, as shown by the cascade plots for each second of bright stimulation (Fig. 3C). The adaptation to naturalistic stimulation allowed LMC output to represent middle and high frequencies, but also increased low-frequency representation of stimulus patterns over time, while photoreceptor output showed little change, leading to dynamic but sub-optimal leveling of the synaptic gain over time (Fig. 3D).

The removal of redundant, *i.e.* low frequency, information is an efficient coding strategy for a system with limited bandwidth, as shown for optimal spatio-temporal filters at high signal-to-noise ratio of bright luminance [10], [11]. On the other hand, if there is an increased encounter of the same input, *i.e.* the pattern repeats, then the system's best coding strategy is to devote an increased frequency representation to these newly encountered features [3], [15], [43]. Here the input was repetitive, and therefore with less sampling of natural statistics, contained certain patterns with uneven frequency spectra. As the R-LMC-R system adapted to these patterns, its encoding was improved further. By keeping account of their previous encounters and using that knowledge to readjust the filtering dynamic, these new statistics were justly represented in the LMC output. Had the R-LMC-R system evolved or adapted only to encode these specific features, they would have been best represented by each of them having an equal frequency representation in the LMC output. Thus, encoding of the stimulus seems sub-optimal as the LMC output never reached that hypothetical equalized distribution.

### Adaptation to repetitive stimulus shows scale-invariance to pattern speed

**Figure 4 pone-0004307-g004:**
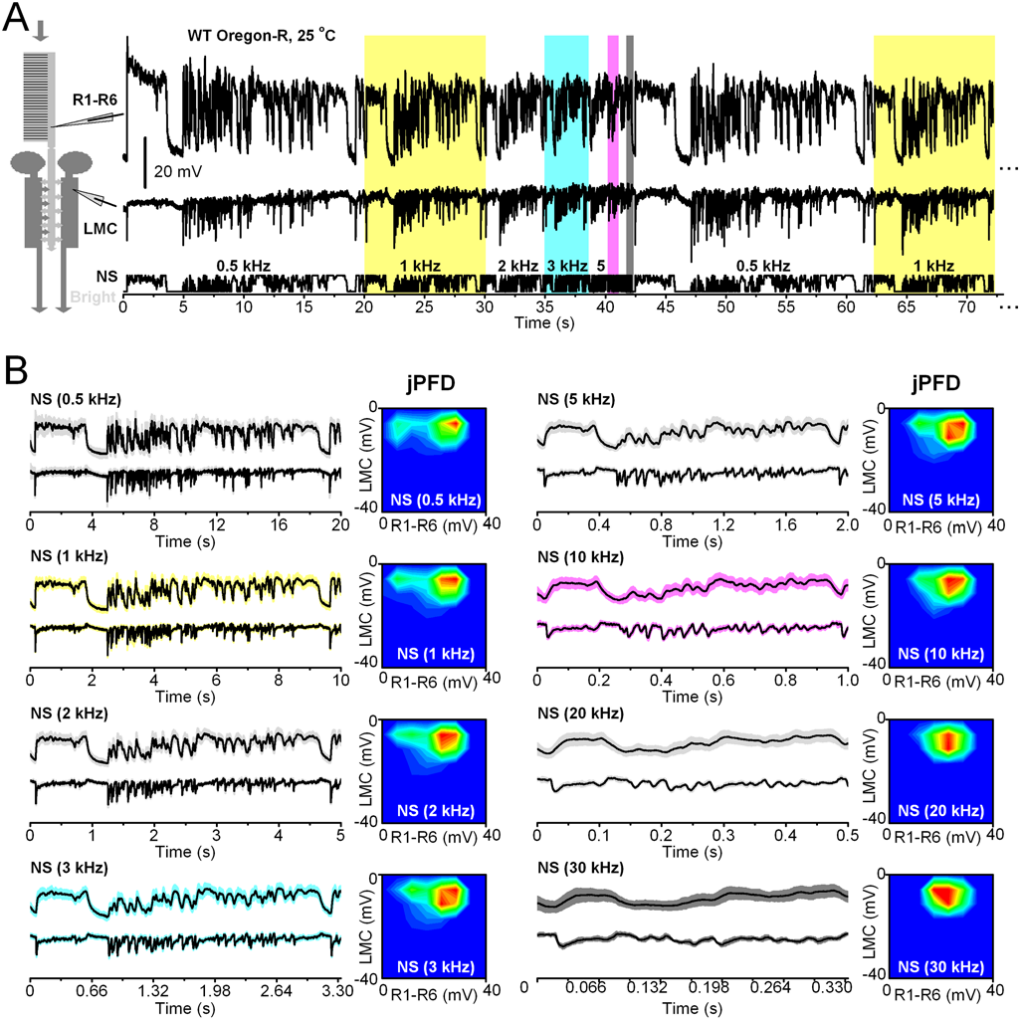
Adaptation to repetitive naturalistic stimulation shows scale-invariance to pattern speed. A. The naturalistic stimulus sequence, NS, repeated at different stimulus playback velocities (bottom trace) and the corresponding intracellular voltage responses of a photoreceptor (top trace) and a LMC (middle trace). The colored sections highlight particular play-back velocities used for the stimulus during this continuous recording (yellow: 1 kHz, 10 s observation window; cyan: 3 kHz, ∼3.3 s window; magenta: 10 kHz, 1 s window; gray: 30 kHz, ∼0.3 s window). B. The time-normalized shapes of the photoreceptor (above) and LMC (below) output emphasize similar aspects of the stimulus, regardless of the used playback velocity (here from 0.5 to 30 kHz). The hot-cold color plots show the corresponding synaptic joint probabilities. Note how the size of the photoreceptor output (horizontal scale) is more reduced than that of the LMC (vertical scale), which remains relatively stable, indicating contrast constancy for all tested playback velocities of stimulation. The changes in the speed of the naturalistic stimulus (attributable to the time-scale invariance of 1/*f* statistics) [24] maintain the power falling within the frequency range of LMC output relatively similar. LMCs can, thus, integrate voltage responses of a similar size for the same stimulus pattern, much irrespective of its speed. Mean±SD shown, n = 7 repetitions.

So far, we have only consider progression of neural encoding within 1 s snapshots, providing a limited view of fast adaptation dynamics. To partially overcome this limitation and to test that our findings were neither biased by the size of the observation window nor the speed of stimulation, we used different playback velocities for naturalistic light intensity series (Fig. 4A). Here, the same stimulus pattern (10,000 intensity values) was repeatedly presented in different periods of time in a single experiment. The playback velocity was increased from the slowest to the fastest, ranging from at 500 Hz for 20 s to 100 kHz for 100 ms, without any delay between repetitions; *cf.* [24]. The resulting voltage responses were sampled with the playback velocity, thereby providing 10,000 samples of data for each pattern speed. These were then used for calculating the joint statistics (Fig. 4B, contour maps).

In a normalized time scale (Fig. 4B), the voltage responses of photoreceptors and LMCs showed striking similarities at different playback velocities of stimulation. This finding implied that neural encoding in the R-LMC-R system possesses considerable scale invariance to the speed and statistical structure of the stimulus [44], [45], [46], particularly for the LMC output. One can see in the contour maps, which show joint probabilities, that the size of the photoreceptor output (horizontal scale) is reduced more than that of the LMC (vertical scale). The LMC output, overall, seemed to withstand speed changes well, indicating contrast constancy for all tested playback velocities of stimulation. Most differences between the photoreceptor and LMC outputs were probably due to their dynamic filtering properties; the photoreceptor output typically low-passing more than the LMC output [25]. Nevertheless, the scale invariance in the LMC output to the stimulus playback velocity probably resulted from the limited integration time [25], and from the self-similarity of time scales in the naturalistic light intensity series [8], [24]. As speeding up, or slowing down, an input with 1/*f* statistics will inherently reallocate a relatively similar power for a given bandwidth, LMCs can integrate responses that utilize their full voltage range.

We tested next that the relevant play-back velocities and observation windows (from 1 s to 100 ms) did not influence the general network adaptation in the R-LMC-R system. An example is shown for 50 kHz playback velocity (Fig. S2). Many experiments were done at 19°C, which slow down adaptation, thereby proving many sample points for which to measure its time constant(s). The overall adaptation dynamics were similar to recordings with slower playback velocities and at higher body temperatures, confirming that our analyses can resolve network adaptation dynamics, at least from the time scales of 100 ms to tens of seconds.

For adaptation in faster time scales, it is useful to consider the frequency range of photoreceptors and LMCs. At 25°C, *Drosophila* photoreceptors and LMCs cannot effectively follow stimulus frequencies higher than ∼150 Hz (Fig. 3C). For 150–200 Hz range, their maximum signal-to-ratios are ∼0.02 (R1–R6s) and ∼0.5 (LMCs); *i.e.* noise >2–50× signal [25]. Notice that due to pooling inputs from six photoreceptors (R1–R6), LMCs have generally a higher signal-to-noise ratio than photoreceptors to naturalistic stimulation [25]. Clearly the fastest transients at the start of bright stimuli can occur in the time-scale of 5–20 ms. However, it is difficult to define when the integration stops and adaptation starts in the WT responses. *Drosophila* LMCs cannot adapt faster than their minimum integration time (5 to 50 ms), which depends on the mean luminance (Fig. 2A) and ambient temperature (*cf.* Fig. S2); see also [36]. Therefore, it is likely that our analyses, which used variable observation windows for testing adaptation dynamics at different luminance levels and temperatures, covered much of the relevant time of network adaptation in the R-LMC-R system for rescaling the LMC output in the natural environment.

### Adaptation changes neural encoding of repetitive stimulus in all light-dark transitions

**Figure 5:**
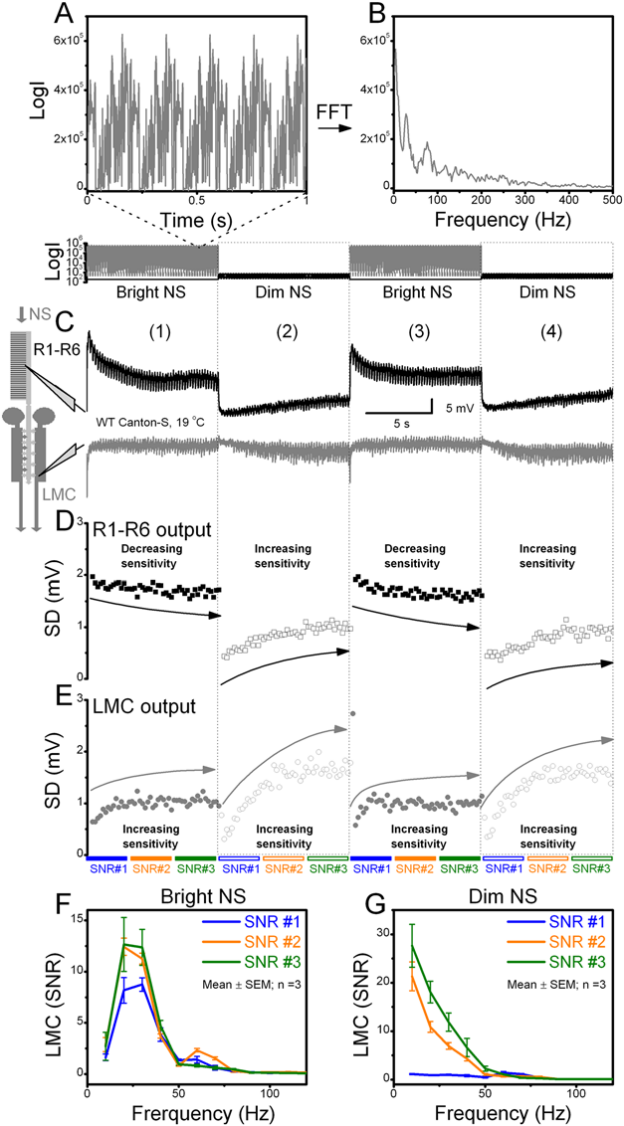
Adaptation improves neural encoding of repetitive naturalistic stimulus in all light-dark transitions. Adaptation sensitizes LMC output over time, rescaling naturalistic contrast stimulus, NS, to a relatively uniform voltage distribution irrespective of the mean luminance and preceding dark/light-adaptation. A–B. Panels show five samples of the same bright stimulus pattern and their frequency spectrum, respectively. C. Typical intracellular voltage responses of a photoreceptor (black) and a LMC (gray) in a WT fly to a 200 ms-long stimulus that was continuously repeated (*cf.* individual traces in Figs. S2). Every 10 s, the stimulus was transiently either brightened or dimmed 10^3^-fold for the next 10 s (dim-bright transitions <1 µs). As expected, the photoreceptor generates larger responses at bright than at dim luminance, whereas the corresponding responses of the LMC show less amplitude variations (*cf.* Fig. 2A). The figure is divided into four columns (1–4) that indicate distinct post-transition periods: (1) from darkness to bright stimuli, (2) from bright to dim stimuli, (3) from dim to bright stimuli, and again (4) from bright to dim stimuli. D. Adaptation in photoreceptor output, shown as SD, was calculated for each 200 ms long response to the stimulus over each post-transition period. Photoreceptor output (mostly due to phototransduction) is desensitized by brightening and sensitized by dimming. The arrows indicate the corresponding adaptive trends. E. Adaptation in LMC output (attributable to synaptic processing) shown as SD, in respect to D. Apart from the transient desensitization (<100 ms), LMC output is sensitized both by brightening and dimming, but this rescaling occurs with different speeds (fast at bright, slower at dim stimuli), similar to LMC output in pre-dark-adapted flies in Fig. 2E. F–G. Signal-to-noise ratio (SNR) of the LMC output for bright and dim stimuli, respectively, calculated from 15 consecutive responses, *i.e.* 3 seconds of data with each response lasting 200 ms. Signal-to-noise ratios are given at different states of adaptation: just after the luminance transition (SNR#1), in the middle of adaptation to given luminance (SNR#2) and in the end of the luminance cycle (SNR#3). Signal-to-noise ratio to the bright stimulus is band-passing and low-passing to the dim stimulus, as predicted for such inputs [11]. In addition, signal-to-noise ratio of LMC output increases with stimulus repetitions, regardless of the luminance level, implying a dynamic increase in the rate of information transfer of naturalistic stimulation by adaptation.

In most experiments, we first dark-adapted the cells for ∼30 s before presenting the stimulus. Thus, it was possible that dynamic optimization of LMC output was just a property of a moderately dark-adapted R-LMC-R system re-sensitizing after being suddenly excited by light increments, and that it would not work with light decrements. To exclude this possibility, we conducted tests in which the stimulus pattern was instantaneously flipped between two different luminance states (<1 µs) every 10 s. Figure 5 shows one such experiment that contained bright and dim stimuli. We took advantage of the relative speed invariance in network adaptation (*cf.* Figs. 4 and S2) by repeating the same pattern (10,000 contrast values) every 200 ms (Figs. 5A–B). Such high sampling provided a high resolution account of the adaptation in the R-LMC-R system between and during the transient luminance transitions.

The main result is that the network adaptation in the time scale >100 ms invariably caused sensitization (Fig 5C–E); *i.e.* it increased the LMC output in all natural light-dark transitions. While we expected that brightening would desensitize the photoreceptor output (black) and dimming would sensitize it (Figs. 5C–D), the general sensitization of the corresponding LMC output (Figs. 5C, E, gray) has not been reported before. LMC output started to increase within 100 ms from the stimulus transition, regardless whether the new light input was transiently brightened or dimmed (note that its contrast distribution remains constant). Importantly, this gain control operated reliably over large intensity range changes (10^3^-fold in Fig. 5), and showed dynamics similar to those seen in the cells after dark adaptation (Fig. 2E). Hence, it appears that network adaptation was striving to generate a relatively uniform LMC output to naturalistic stimulation, irrespective of the desensitization/sensitization dynamics in phototransduction following light- or dark-exposure.

### Adaptation increases signal-to-noise ratio of LMC output in all light-dark transitions

The arguments of efficient neural encoding are about improving the statistics of neural responses (signals) to represent relevant information about the stimulus. However, it was still possible that the boosted LMC output to repetitive stimulation resulted from an increase in noise. To rule out this possibility, we further used these recordings to approximate changes in the signaling performance of the R-LMC-R system (Figs. 5F–G). We calculated the signal-to-noise ratio of LMC output to the bright and dim stimulus at different states of adaptation at 0–3 (SNR#1), at 3.5–6.5 (SNR#2) and at 7–10 (SNR#3) seconds after each luminance transition. The first responses at new luminance were disregarded because of their large variability, while the use of brief data segments (3 s) minimized the possibility that differences in the signal-to-noise ratio estimates would result from the recordings deteriorating. Notice, however, that neural signal-to-noise ratios [24] are underestimates, as the noise, *i.e.* the difference between the signal (mean response) and individual responses, contains adaptation and stochastic variability. Therefore, although each signal-to-noise ratio was calculated using the same amount of data (15×200-points) and averaged over three separate luminance sections to minimize bias, these enable only relative comparisons.

Nonetheless, we found that the LMC's signal-to-noise ratio to bright stimulation was band-passing (Figs. 5F) and low-passing to dim stimulation (Figs. 5G), in concordance with the general concept of optimal coding for such inputs [11]. Furthermore, the signal-to-noise ratio of LMC output increased with repeated stimulus presentations at both luminance levels, even after the adaptive trends had mostly subsided (SDs, Fig. 5E). The fact that SNR#3 is larger than SNR#1-2, during both the dim (Figs. 5F) and bright (Fig. 5G) stimulation, provides strong evidence that the signaling performance of the system improves continuously. Thus, the increase in the LMC's voltage responses to the stimulus could not result from increase in noise, but instead it resulted from increase in signal. Therefore, these findings support the tenet that by improving neural encoding, adaptation increases the rate of information transfer to naturalistic stimulation in the R-LMC-R system.

### Adaptation improves neural encoding of repetitive stimulus over time

Together, our results (Figs. 2–5) imply a dynamic improvement in the amplitude and frequency representations of naturalistic stimuli in the voltage responses of LMCs increased with “flattened” and “whitened” output probabilities, respectively. The time scale of this redistribution depended on luminance but occurred within seconds, and thus was probably too fast for trafficking or expressing ion-channel proteins in LMCs [47]. Because LMCs output remained relatively constant at different luminance level, we consider voltage-dependent changes in the membrane impedance an unlikely explanation for adaptation; but see [15], [48], [49]. With brightening stimulation the total amplitude range of LMCs increased only by ∼25% (best cells: from ∼30 mV to ∼40 mV, Fig. 2A), while the time-constant of adaptation varied 4-fold (Fig. 2E). Instead, it is far more likely that the improved transmission properties of LMCs reflected a gradual increase in histamine-gated chloride conductance [37].

There are two obvious mechanisms that could modify the “input”-conductance of LMCs: (i) an increase in the open-probability of the histamine receptors, or (ii) an increase in the histamine release probability, *i.e.* ligand concentration. Both of these mechanisms were likely to be continuously adjusted by the dynamic equilibrium between light- and feedback-mediated conductances, as the photoreceptor voltage was driven up and down by light changes. In the companion paper [36], we dissect these hypotheses by separately manipulating the strength of the synaptic feedforward (R-LMC) or feedback (L2-R) pathways, and show that both of these mechanisms are indeed necessary for improving the temporal representation of naturalistic stimuli in the R-LMC-R system of the *Drosophila* eye.

## Discussion

This study was aimed at understanding the dynamics of retinal adaptation to temporal contrast patterns in the *Drosophila* eye. We examined the transfer of repeated naturalistic contrast patterns in the R-LMC-R system that consists of photoreceptors and interneurons that co-process visual information from one point in visual space through extensive feedforward and feedback connections [25], [32]. The results show that network adaptation makes neural encoding of visual information in the R-LMC-R system more effective over an extensive time-span (>100 ms to seconds) by boosting under-represented signals in LMC output. The fact that network adaptation takes place over the tested intensity range (4 log-units in Figs. 2–3 and S1), with different stimulus patterns and pattern speeds (Figs. 4 and S2), in naturalistic light-dark transitions (Figs. 2–3 and 5) and at different temperatures (Figs. S1 and S2) highlights its physiological significance for efficient neural encoding in the R-LMC-R system.

In the following discussion, we consider the advantages of using *Drosophila* as a model to study neural information processing in the retinal circuits, and highlight some possible benefits of network adaptation for the fly vision. We then briefly discuss the different ways adaptation has been studied previously in action potential and graded potential systems and the basic assumptions for testing the ideas of efficient neural encoding. Finally, we comment on the limitations of our findings.

### *Drosophila* is a good model to study retinal adaptation

The natural advantages of using *Drosophila* for studying retinal adaptation are an existing circuit reconstruction from electron microscopy [32], [35], the ability to modify the network using molecular methods and the possibility to quantify visual behavior [50]–[52]. An added benefit of the small *Drosophila* eye for synaptic electrophysiology is that its photoreceptors have relatively short axons, yet the somatic membrane has high resistance [22], which provides a high length constant. Thus, via high-quality intracellular recordings from photoreceptor somata, it possible to identify echoes of on-going activity in their synaptic terminals *in vivo* [25]. Such activity is negligible in conventional sharp microelectrode recordings from photoreceptors of bigger flies that are leakier [53] and have longer axons.

### Network adaptation improves neural encoding and may facilitate image constancy for changing conditions

The R-LMC-R system relies upon complex dynamic nonlinear interactions (Figs. 2B–C, contour maps) to translate vast environmental luminance changes into voltage responses of limited size and speed. Similar to blowfly [17], [20], we found in *Drosophila* that phototransduction performs a logarithmic compression of the naturalistic light intensity series while photoreceptor-LMC interactions work toward contrast constancy by normalizing this signal (Fig. 2A). Importantly, our findings further showed that adaptation uses previous encounters with the local stimulus statistics, *i.e.* their temporal structure, to improve (widen) the LMC output continuously (Figs. 5F–G). What does this mean for fly visual behavior?

The *Drosophila* compound eye - with its ∼750 ommatidia – captures a scene only with relatively low spatial resolution [50], [54] and is expected to produce a “blurred” neural image for the brain [5]. However, when the image quality at each pixel (Fig. 1A) is continuously adjusted to its light input, the collective neural image presented to the brain improves. As in digital processing of photographic images [40], [55], neural images can be made clearer (or stand out better against the background) by equalizing their contrast and frequency spectra (Figs. 1B–C “flattening” and “whitening”; Figs. 2C–D and 3C–D, respectively) around local values. As this occurs dynamically across the compound eye, it is likely to produce a parallelly edited neural “movie” for the fly brain that may facilitate tracking of visual objects of interest [56], [57] or flow processing [58] in changing conditions.

Interestingly, the recent study by Brinkworth *el al.* [59] from blowfly photoreceptors identified that the process of target detection begins already at these cells; see also [60]. By analyzing their intracellularly recorded output to a stream of natural image sequences, they could show that temporal processing by photoreceptors, even without any spatial interactions, significantly improved the discrimination of small targets. They then applied a modified van Hateren-Snippe model of a blowfly photoreceptor [6], [61] to show that such enhancement of target salience could be explained by temporal nonlinear dynamics. Considering the similar synaptic layout of the fly compound eyes, and the limited visibility of the somatic photoreceptor recordings to the synaptic interactions in the lamina in the blowfly preparation (see above), perhaps at least part of the improvement for target salience was due to network adaptation in the R-LMC-R system, as we have shown here.

### Adaptation in action potential and graded potential systems

In spiking systems, one can simplify complex input-output transformations into single variable mapping operations for spike rates, intervals or times, by making the assumption that action potentials are uniform (or digital) carriers of information; but see also [62], [63]–[66]. In many sensory systems, when stimulus intensity, contrast or velocity values are mapped against these variables, adaptation dynamics of neural spike patterns can be successfully mimicked with simple linear-nonlinear (LN) models, *e.g.* a temporal filter followed by a static transfer function. In some systems, the spike patterns have been further shown to adapt to local characteristics of the stimulus statistics, indicating efficient coding, *e.g.* [16], [18], [44], [45], [67]–[71].

The same approach of reduction of variables has been also applied to a graded potential system. In the pioneering study of efficient coding, it was assumed that the maximum amplitudes in the graded voltage responses of LMCs encode luminance contrast values [4]. However, because of the complex information processing in the R-LMC-R system (Figs. 2–5), neither this assumption nor the resulting mapping is accurate; see also [11], [17], [25], [27], [72]. The assumption is inaccurate because static singularities, such as the maximum amplitude of a response, correlate only weakly even with stationary contrast stimuli [27], [72]. Because of network adaptation, the nonstationary R-LMC-R system shows extensive complexity (Fig. 2B–C). The same maximum amplitude can be evoked by a shorter pulse of higher contrast [27], [72], and therefore carries relatively little contrast information in comparison to their true response waveforms, or even to the rate of rise or fall of the responses [25], [28], [62], [72]. The mapping is inaccurate, because an arbitrary static nonlinear mapping function can be selected within a large range of maximum responses by changing the duration or interstimulus interval of the contrast pulses - which control the integration of voltage responses [27], [72]. However, none of these mapping functions can adequately define the complex LMC output to global or local contrast distributions. See also [17].

### Why is network adaptation not static optimization?

For static optimization the original case was made that the maximum amplitudes of the LMCs' voltage responses map the cumulative distribution of natural contrasts (or the global statistics) so that all response levels are used equally often [4]. Such optimal mapping required that the synaptic gain function, *i.e.* the “characteristic curve”, would remain unaffected by the R-LMC-R system's state of light adaptation [38]. For the given contrast pulses at different light backgrounds, the “characteristic curve” mapped the evoked maximum responses of photoreceptors to those of LMCs, although these peak values have no causal time relationship [27], *cf.* Fig. 4A. Notably, even if one uses peak responses for a “ballpark-estimate” of contrast coding, optimal mapping would always be affected by the R-LMC-R system's adaptation state, unless the signal-to-noise ratio was the same at all luminance levels. The fact that this is not the case is a good reason for altering the frequency response, particularly increasing low frequencies at dim and high frequencies at bright conditions [11], [17].

Since then, experimental and theoretical work has shown that the neural code of LMCs is *dynamic* and *continuous*, and it adapts to changing statistics [11], [14], [17], [21], [27], [73]–[75]. Our results here build on these studies by identifying network adaptation as a neural mechanism for adjusting the R-LMC-R system's throughput. As the synaptic gain is continuously adjusted to the changing statistics by adaptation (Figs. 2–5), gradual changes in both the voltage and frequency range of LMCs can improve the neural representation of contrast information. Of course in real life, neural encoding of natural stimuli happens both in space and time, wherein the animal's sensing and actions are in a closed-loop with the environment. Furthermore, apart from temporal contrast, we know relatively little about how the R-LMC-R system contributes in transmitting other features in the natural scenes, such as colour, line elements, orientation or motion, to the fly brain, or how the same network structures may be used for different processing tasks simultaneously. Thus, such real life encoding of visual information from natural scenes is likely to be even more sophisticated than what is currently possible to measure in laboratory conditions [17], [57], [59], [67], [76]–[86].

The companion paper [36] that focuses on the mechanisms of network adaptation discusses some of these issues further.

## Materials and Methods

### Flies

Wild type (WT) Oregon-R and Canton-S strains were used for recordings; they also provided the controls for the genetic dissection of the R-LMC-R circuitry in Part II [36]. Flies were reared on standard medium at 18°C in 12∶12 light∶dark cycle [87] and females were selected for electrophysiological experiments 4 days after eclosion. In both of these WT fly stains, adaptation in the R-LMC-R system to repeated naturalistic light intensity patterns occurred alike.

### *In vivo* electrophysiology

Flies were prepared *in vivo* [22] and intracellular voltage responses of blue-green-sensitive R1–R6s and LMCs were recorded separately using sharp quartz or borosilicate microelectrodes [22], [25] (Sutter Instrument Co, USA) of resistance 120–200 MΩ. The intraelectrode solution was 3 M KCl for photoreceptor experiments, or 3 M potassium acetate with 0.5 mM KCl for LMCs to sustain their chloride battery [37]. Responses were amplified by SEC-10L (NPI Electronic, Germany) in current-clamp mode using ∼15 kHz switching rate and low-pass filtered with light stimuli at 1.5 kHz (Kemo VBF8, UK). During the experiments, the flies were immobilized within a brass fly-holder, placed on a Peltier-device [23]. The ambient air temperature was maintained by air conditioning at 19.0±0.5°C, whereas the head-temperature of the flies was set to 25±0.5°C (WT Oregon-R) or 19.0±0.5°C (WT Canton-S) by a feedback-controlled Peltier-device [23]. 21.5–25.5°C is the preferred temperature range of both WT Canton-R [88], [89] and *ort* ^6^ mutants [89] used in Part II [36]. WT Canton-S flies were tested at 19°C, as this data will be compared with the transgenic expression of the temperature-sensitive mutant *shibire^TS1^* in Part II [36].

### Intracellular voltage responses and selection criteria

R1–R6 photoreceptors depolarize and LMCs hyperpolarize to light, making their identification easy [25], [90]. In contrast, the differences in responses between different LMC subtypes are subtle. The largest and the most central of LMCs, L1 and L2, share their synapses, whereas synaptic input to more proximal L3 cells is less prominent [35]. In *Calliphora*, L1 and L2 generate virtually identical responses in lamina. The responses of L3 are more hyperpolarized, having the largest off-transients [91]. We made no attempt to identify different LMC subtypes, but because L1 and L2 occupy the largest volume it is likely that most recordings were in them. It is also possible that some responses were from processes of amacrine cells that share histaminergic input with L2 and L1 cells [25], [92]. Nevertheless, because the waveforms of hyperpolarizing responses to the same naturalistic stimulus pattern in *Drosophila* lamina have rather similar characteristics, these all were pooled [25].

To prevent poor penetrations or electrodes biasing our analysis, only stable high quality recordings were used. Such photoreceptors had resting potentials in darkness <−60 mV and maximum responses to the tested bright stimulus >35 mV. Their dark-adapted impulse responses were >40 mV. For the selected LMCs, the resting potentials were <−30 mV and maximum responses >15 mV. Note that here we included LMCs with smaller amplitudes than in our previous study [25] because the response dynamics of these cells (Figs S1C–H), when normalized, were practically identical to those of cells with the largest response ranges (30–45 mV, Figs. S1A–B), and their signal-to-noise ratio to the repeated stimulus were similarly high. Thus, apart from reflecting the recording quality, the size of the responses may also reflect their subcellular locations. For instance, responses in LMC somata require back-propagation and therefore may be smaller than those at the synaptic zones. As the somata make larger microelectrode targets than the synaptic zones, most recordings should be somatic. More details of the electrophysiology in the *Drosophila* eye are in [22], [25].

With these criteria, 83.3% of WT Oregon-R LMCs (90/108 experiments) at 25°C and 81.8% WT Canton-S red LMCs (90/110 recordings) at 19°C, showed increasing adaptive trends in their responses to the repeated stimulation, *i.e.* the LMC output to the same naturalistic light contrast pattern was boosted over time. Thus, the findings indicate that network adaptation happens independently of the fly strain or temperature. Figure S1 summarizes the statistical analysis of 54 recordings from the LMCs of WT Oregon-R at 25°C. In the best series (*cf.* Fig. S1A), the adaptive trends always behaved consistently, becoming faster with brightening stimulation (Fig. S1B). Owing to their high reliability (Fig. S1C), only the recording series with the largest voltage responses (>30 mV to the given stimulus pattern at all luminance levels) were used in Figures 2–3. We had two complete recording series from single LMCs at seven different luminance levels (one of them shown in Fig. S1A). Both of these series showed highly similar adaptation at different luminance levels, but their maximum voltage ranges were ∼40 and ∼45 mV, respectively. However, for not to bias the adaptation metrics and to keep the probability functions (see below) free of normalization/rescaling (for easy assessment), we displayed the photoreceptor and LMC output on the same 50 mV voltage scale and used only recordings that were collected through identical light adaptation protocols. All seven photoreceptor series used in Figs. 2–3 were adapted to the same seven luminance levels in the same order as the representative LMC series. This procedure, furthermore, kept their expected signal-to-noise ratios roughly comparable [93].

The presented results (Figs. 2–5) are general and occur also in recordings with smaller amplitudes [36]; each result is supported by stable recordings in at least three different cells.

### Light stimulation and data collection

NS patterns were selected from the van Hateren natural-stimulus-collection, http://hlab.phys.rug.nl/archive.html [17], and we used two different LED-based systems to play them back to the flies. The distinctive adaptive behaviors of photoreceptors and LMCs remained unchanged regardless of the stimulator used. The responsiveness of the cells was also tested with different naturalistic stimulus sequences (data not shown). Again, this had little effect of their adaptation trends.

R1–R6 photoreceptors and LMCs were stimulated by light from high-intensity green LEDs (Marl Optosource, UK, peak emission: 525 nm). The light stimulation was delivered by a randomized fiber optic bundle, secured on a Cardan arm system. This arrangement enabled free positioning of the light source with equal distance to the eye with the LED output subtending 5°, as seen by the fly. The brightness of stimulation was changed by placing neural density filters on the light source. This way the contrast of the naturalistic stimulus sequence (*c* = Δ*I*/*I*) remained constant at all tested stimulus conditions. As the variance of stimulation increased with mean intensity, a simple adaptation mechanism, such as intracellular pupil is insufficient to bring LMCs back to their coding range. Thus, a more complex response is required for the rescaling of the output (*cf.* Figs. 2–5). The intensity range covered 4 log-units [24], [25] from ∼600 to ∼6×10^6^ photons/s (*I*_0_). Figures show results for dim- (1,850), medium- (60,000) and bright-light (1.85×10^6^ photons/s).

For Figures 2–3, the cells were first dark-adapted for a minimum of 30 s. The cells were first tested with a dim stimulus before processing to brighter stimuli. Between the different luminance levels, the cells were dark adapted for 30 s. However, we found that the duration of dark-adaptation had relatively little effect for the brightness-dependent adaptive trends of the LMC output to repeated naturalistic stimuli, as shown in Figures 4–5 and S2. The fast component of their adaptation was nearly instantaneous, followed by the slower sensitization to the stimulus of new luminance. Typically, the stimulus and response were sampled at the rate of the playback velocity used for the stimulus, or at 1 kHz. The data was often re-sampled/processed off-line at 1 kHz for the analysis. Stimulus generation, data acquisition and analysis were performed by Matlab interface BIOSYST [22], [24].

For Figure 5, naturalistic stimulation was delivered via a randomized Y-fiber optic bundle, in which the common-end pointed to the centre of the cell's receptive field and the two arms received separately either the dim or bright stimulus (from two LED drivers – their light outputs were pre-scaled by neural density filters). Two LED drivers alternated, generating either dim or bright sequences of the identical contrast distribution in 10 s intervals.

### Probability density and joint probability functions

We measured the probability density (PDF) and joint probability density (jPDF) functions of the photoreceptor and LMC output to the repeated stimulation by mapping one-to-one their corresponding voltage values at 1 ms time-resolution over the evolution of the experiments. The occurrence of single (R1–R6 or LMC) and paired (R1–R6 and LMC) voltage values were counted and given as probabilities, either for the duration of the experiment (*cf.* Fig. 2B) or for each 1-s-long stimulus repetition (observation window, *cf.* Fig. 2C). Most calculations correlated seven individual recording series of WT photoreceptors to the best corresponding LMC series using 50 mV absolute voltage scale with 3.3 mV resolution. Although the probabilities varied from one luminance to another and with time, the results were similar within each WT genotype at a given luminance and moment of time. Therefore, the probabilities were presented as the means of such distributions. The synaptic delay (<1 ms) had no effect on the shown jPDFs.

Because we used observation windows, ranging 0.1–1 s, that were smaller than the relatively slow adaptation time-courses studied (τ = 1–20 s), PDFs, jPDFs and spectra (see below) approximate stationarity. These statistical metrics, thus, provide reliable estimates of synaptic gain changes from one observation window to another, in which all sampled voltage values were used for the calculations.

### Adaptive trends in continuous recordings

As a simple measure of a changing voltage distribution, we quantified adaptive trends in a cell's responsiveness to repeated stimulation. Here, we took the standard deviation (SD) of the first 20 voltage responses, using the last 8,000 samples (time points) of each response (10 kHz sampling). If the system's sensitivity to the stimulus increases, so as to code more efficiently, the distribution of its output, and thus its SD, should widen over time: *c.f.* “flattering” and “whitening” (Figs. 1B–C, respectively). This is indeed the case for LMC output, for which signal-to-noise ratio increases over stimulation, while the signal-to-noise ratio of the naturalistic stimulus remains constant (Fig. 5).

For each genotype, the adaptive trends of their photoreceptors and LMCs were grouped separately for each tested temperature and brightness. To make the comparison of these groups immune to different amplitudes of individual recordings (see Fig. S1), we used the following procedure. The adaptive trend of each individual recording was normalized by the SD value of its first response to the stimulus (at the 1^st^ s), giving the relative change in a cell's voltage output over time. Normalized trend = (SD_n_−SD_1_)/SD_1_, where SD_n_ is calculated from individual responses, n (using 201–1,000 ms), and SD_1_ from the 1^st^ response to the stimulus. The mean and its standard error (SEM) of the normalized trends for each group of cells were then adjusted to mV-scale by multiplying them with the measured average SD-value of each population at the first second (Fig. 2E).

### Frequency Analysis

The throughput of the R-LMC-R system adapts during repetitive stimulation. We quantified these changes in frequency domain by comparing corresponding frequency spectra of photoreceptor and LMC outputs in WT and mutant flies for the first 20 s for each second of stimulation. This procedure also kept mV as the unit, further facilitating comparisons between the raw data and SDs. To obtain both the suitable range and reliability for the spectral estimates, the following procedures were used. The data was re-sampled at 1 kHz and the power spectrum 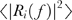 for each 1 s long response, *R*_i_, was calculated using a 500-point Blackman-Harris window with 50% overlap, *i.e.* n = 3 spectral samples, where | | denotes the norm and 〈 〉 the average over the different stretches as in [22]. The square root of the power spectra then gave the mean frequency spectrum 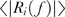 for each response to the repeated stimulus, from 2 to 500 Hz with 2 Hz resolution (Figs. 3C).

We also analyzed the frequency spectra of photoreceptor and LMC output for the stimulus of different luminance levels (dim, middle and bright). Here, all the mean frequency spectra for each light level were averaged, using all the data from the first to 20 voltage traces, *i.e.* n = 60 spectral samples. See Fig. 3A.

The ratio between the corresponding mean LMC and photoreceptor frequency spectra gave the synaptic gain (Figs. 3B and 3D) function for each stimulus presentation or for each light level.

Signal-to-noise ratio (Fig. 5), SNR(*f*), is the ratio between the signal 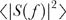 and noise 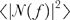 power spectra, | | and 〈 〉 as above. The signal power spectrum was calculated from the mean voltage response, using 15 consecutive 200 ms long responses to the repeated stimulus with 1 kHz sampling. The noise power spectra was calculated from the corresponding noise traces, *i.e.* the differences between individual responses and signal. Again, these data chunks were divided into 50% overlapping stretches and windowed with a Blackman-Harris 4-term window, each giving three 100-points long samples. These were averaged to improve the estimates.

In all cases, Matlab's Fast Fourier Transform (FFT) algorithm was used to calculate the power spectra.

## Supporting Information

Figure S1 Voltage output of WT Oregon-R LMCs to repeated naturalistic stimuli (NS) at different luminance. Voltage output of WT Oregon-R LMCs to repeated naturalistic stimuli (NS) at different luminance. LMC output behaves systematically, although the size of the responses varies greatly from one cell to another. A. Intracellular voltage responses of a single exceptionally stable LMC, measured to a repeated 1-s-long NS at seven different brightness-levels, each 0.5 log intensity units apart. Responses to bright (dark green), middle (red) and dim (blue) NS highlighted; responses to intermediated light levels are shown in black. B. The SD of these responses (*i.e.* adaptive trends; each point calculated from 800 ms long data-sections) to the repeated NS pattern increases over time, but their rate of rise depends on the luminance of the NS. C. The adaptive trends of all data to dim, middle and bright NS at 25°C. Notice the large variation in the size of the voltage responses. The best data (blue, red and green) is used for Figure 2 in the main paper; the rest of the data is shown in gray-scale. D. The mean±SEM of the adapting trends using all data. E. The mean±SEM of the adapting trends using only the best data. The trends are well fitted with single exponentials. F. The increase in response size (or sensitivity) of the best data over the repeated dim, middle and bright NS given as percentage (mean±SEM); the trend for each experiment is normalized by its first value. G. The normalized trends of all the recordings. H. The statistics of the normalized trends, using all data (mean±SEM). (F–H) Sensitivity = 100*(SD_n_−SD_1_)/SD_1_; where SDn is calculated from individual responses, n (using 201–1000 ms), and SD_1_ from the 1st response to NS. The fits in E are done using the average SDs of the best recordings. If instead the traces included in the average curves are fitted separately, we obtain (mean±SEM): τ_bright_ = 1.51±0.19 s (n = 4); τ_middle_ = 4.41±0.96 s (n = 7); τ_dim_ = 6.27±2.94 s (n = 4). Furthermore, for all the traces that allowed adequate fitting we obtain: τ_bright_ = 1.91±0.21 s (n = 7); τ_middle_ = 4.20±0.49 s (n = 19); τ_dim_ = 4.26±1.26 s (n = 11). Here, only the means of τ_bright_ and τ_middle_ differ significantly (p<0.007; ANOVA, Bonferoni test). This seems attributable to invariable quality of the recordings at dim NS. Unlike the best series that covered the whole brightness range (such as in A), often individual recordings at dim NS were less stable, capturing only small and noisy responses, whose trends (G) were highly variable and fitting unreliable.
(1.17 MB TIF)

Figure S2 Photoreceptor and LMC outputs to repeated NS is independent of the observation window and the speed of stimulation. A 10,000-points-long NS pattern was repeatedly presented to a WT Canton-S photoreceptor and LMC at 50 kHz (200 ms observation window; i.e. the duration of each input and output) at 19°C. A. Four first voltage responses of a photoreceptor (n1–n4) evoked by the repeated NS at middle luminance. B. Photoreceptor output plotted over the duration of the experiment; the voltage range of R1–R6 is reduced over tens of seconds as the photoreceptor adapts to the input statistics. C. This drop in the overall sensitivity is well fitted with two-exponentials. Photoreceptor adaptation has a similar decaying trend as seen with the 10 kHz NS (*cf.* Figs. 2E). D. The first four voltage responses of a LMC evoked by the same NS. E. LMC output over the duration of the experiment; the voltage range of the LMC increases gradually as the R-LMC-R system adapts to the stimulation. F. LMC output is boosted similar to 1 s NS (*cf.* Fig. 2E). The increase in the overall sensitivity of the LMC is fitted with two-exponentials; the dominant one having a slightly slower value to data with 1 s window (cf. Figs. 2E), possibly because of the cooler temperature. In A and E, the relative long delays (20 ms) in the first responses is attributable to the phototransduction dead-time, and the inability of the photoreceptor and LMC to respond to fast changes in the NS, attributable to their relative slow integration times. Sensitivity = 100*(SD_n_−SD_2_)/SD_2_; where SDn is calculated from individual responses, n (using 1–200 ms), and SD_2_ is from the 2nd response to NS (note in D that ∼100 ms after the first light onset, the responses already increase).
(0.59 MB TIF)
