# Supplementary figures and images for: Network Adaptation Improves Temporal Representation of Naturalistic Stimuli in Drosophila Eye: I Dynamics
Source: PLoS One. 2009 Jan 30;4(1):e4307. doi: 10.1371/journal.pone.0004307 (PMC2628724; doi:10.1371/journal.pone.0004307)

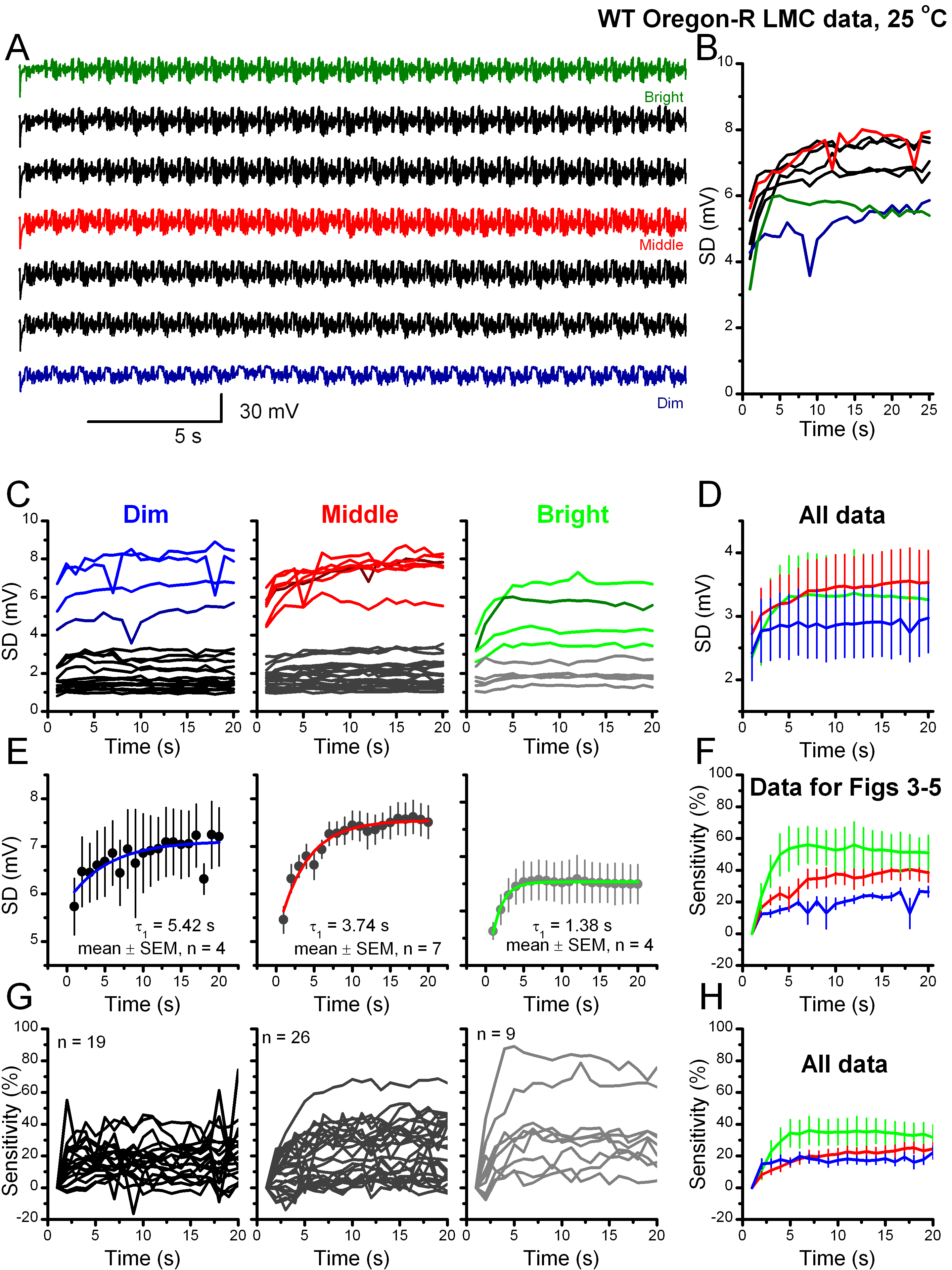

Supplement: Figure S1 — Voltage output of WT Oregon-R LMCs to repeated naturalistic stimuli (NS) at different luminance. Voltage output of WT Oregon-R LMCs to repeated naturalistic stimuli (NS) at different luminance. LMC output behaves systematically, although the size of the responses varies greatly from one cell to another. A. Intracellular voltage responses of a single exceptionally stable LMC, measured to a repeated 1-s-long NS at seven different brightness-levels, each 0.5 log intensity units apart. Responses to bright (dark green), middle (red) and dim (blue) NS highlighted; responses to intermediated light levels are shown in black. B. The SD of these responses (i.e. adaptive trends; each point calculated from 800 ms long data-sections) to the repeated NS pattern increases over time, but their rate of rise depends on the luminance of the NS. C. The adaptive trends of all data to dim, middle and bright NS at 25°C. Notice the large variation in the size of the voltage responses. The best data (blue, red and green) is used for Figure 2 in the main paper; the rest of the data is shown in gray-scale. D. The mean±SEM of the adapting trends using all data. E. The mean±SEM of the adapting trends using only the best data. The trends are well fitted with single exponentials. F. The increase in response size (or sensitivity) of the best data over the repeated dim, middle and bright NS given as percentage (mean±SEM); the trend for each experiment is normalized by its first value. G. The normalized trends of all the recordings. H. The statistics of the normalized trends, using all data (mean±SEM). (F–H) Sensitivity = 100*(SDn−SD1)/SD1; where SDn is calculated from individual responses, n (using 201–1000 ms), and SD1 from the 1st response to NS. The fits in E are done using the average SDs of the best recordings. If instead the traces included in the average curves are fitted separately, we obtain (mean±SEM): τbright = 1.51±0.19 s (n = 4); τmiddle = 4.41±0.96 s (n = 7); τdim = 6.27±2.94 s (n = 4 [file pone.0004307.s001.tif]

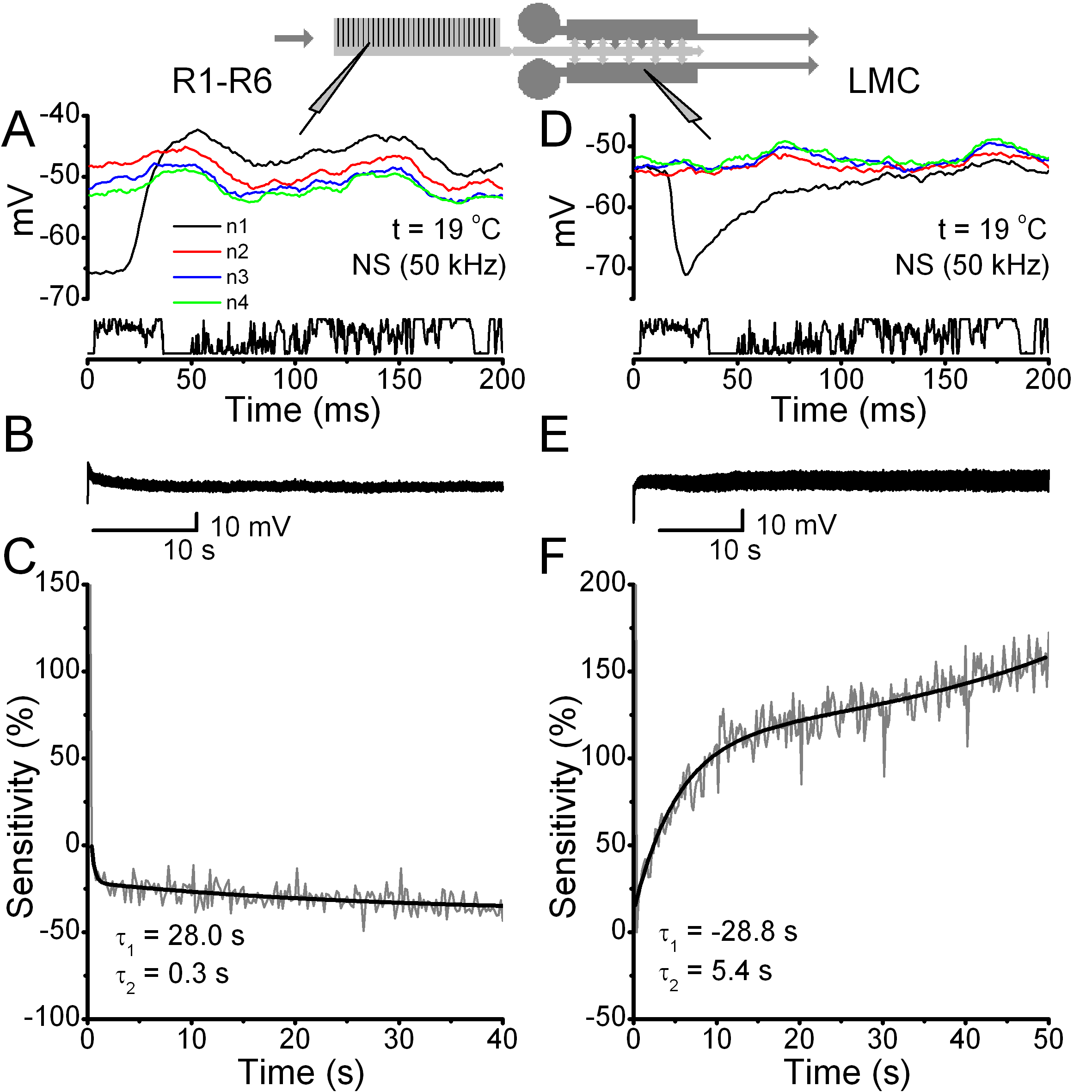

Supplement: Figure S2 — Photoreceptor and LMC outputs to repeated NS is independent of the observation window and the speed of stimulation. A 10,000-points-long NS pattern was repeatedly presented to a WT Canton-S photoreceptor and LMC at 50 kHz (200 ms observation window; i.e. the duration of each input and output) at 19°C. A. Four first voltage responses of a photoreceptor (n1–n4) evoked by the repeated NS at middle luminance. B. Photoreceptor output plotted over the duration of the experiment; the voltage range of R1–R6 is reduced over tens of seconds as the photoreceptor adapts to the input statistics. C. This drop in the overall sensitivity is well fitted with two-exponentials. Photoreceptor adaptation has a similar decaying trend as seen with the 10 kHz NS (cf. Figs. 2E). D. The first four voltage responses of a LMC evoked by the same NS. E. LMC output over the duration of the experiment; the voltage range of the LMC increases gradually as the R-LMC-R system adapts to the stimulation. F. LMC output is boosted similar to 1 s NS (cf. Fig. 2E). The increase in the overall sensitivity of the LMC is fitted with two-exponentials; the dominant one having a slightly slower value to data with 1 s window (cf. Figs. 2E), possibly because of the cooler temperature. In A and E, the relative long delays (20 ms) in the first responses is attributable to the phototransduction dead-time, and the inability of the photoreceptor and LMC to respond to fast changes in the NS, attributable to their relative slow integration times. Sensitivity = 100*(SDn−SD2)/SD2; where SDn is calculated from individual responses, n (using 1–200 ms), and SD2 is from the 2nd response to NS (note in D that ∼100 ms after the first light onset, the responses already increase). (0.59 MB TIF) [file pone.0004307.s002.tif]
